# Effect of *Piriformospora indica*-Induced Systemic Resistance and Basal Immunity Against *Rhizoctonia cerealis* and *Fusarium graminearum* in Wheat

**DOI:** 10.3389/fpls.2022.836940

**Published:** 2022-04-14

**Authors:** Liang Li, Nannan Guo, Yu Feng, Mengmeng Duan, Chunhui Li

**Affiliations:** School of Chemical Engineering and Technology, Hebei University of Technology, Tianjin, China

**Keywords:** *Piriformospora indica*, differentially expressed gene (DEGs), sharp eyesspot, root rot, reprogram

## Abstract

Wheat is among the top 10 and most widely grown crops in the world. However, wheat is often infected with many soil-borne diseases, including sharp eyespot, mainly caused by the necrotrophic fungus *Rhizoctonia cerealis*, and Fusarium head blight (FHB), caused by *Fusarium graminearum*, resulting in reduced production. *Piriformospora indica* is a root endophytic fungus with a wide range of host plants, which increases their growth and tolerance to biotic and abiotic stresses. In this study, the capability of *P. indica* to protect wheat seedlings against *R. cerealis* and *F. graminearum* was investigated at the physiological, biochemical, and molecular levels. Our results showed that *P. indica* significantly reduced the disease progress on wheat caused by *F. graminearum* and *R. cerealis in vivo*, but not showed any antagonistic effect on *F. graminearum* and *R. cerealis in vitro*. Additionally, *P. indica* can induce systemic resistance by elevating H_2_O_2_ content, antioxidase activity, relative water content (RWC), and membrane stability index (MSI) compared to the plants only inoculated with *F. graminearum* or *R. cerealis* and control. RNA-seq suggested that transcriptome changes caused by *F. graminearum* were more severe than those caused by *R. cerealis.* The number of differentially expressed genes (DEGs) in the transcriptome can be reduced by the addition of *P. indica:* for *F. graminearum* reduced by 18% and for *R. cerealis* reduced 58%. The DEGs related to disease resistance, such as WRKY and MAPK, were upregulated by *P. indica* colonization. The data further revealed that the transcriptional resistance to *F. graminearum* and *R. cerealis* mediated by *P. indica* is quite different.

## Introduction

Wheat is an economically important crop worldwide and approximately 35∼40% of the world’s population take wheat as the main food. As the world’s largest producer and consumer of wheat, the stability of wheat production in China is of great significance to guarantee not only national but also world food security. However, one of the major problems of these crops is the ecological loss caused by a variety of diseases, which has caused more than 1 billion yuan (RMB) of losses per year from 2005 to 2008 (Chen et al., 2008). Wheat sharp eyespot is a serious disease caused by *Rhizoctonia cerealis*, infecting both the stems and sheaths of wheat plants, blocking the transportation of nutrients required for growth (Chen et al., 2008; Wang et al., 2021). *Fusarium graminearum* is another necrotrophic fungus that can cause root rot and head blight in wheat around the world (Osborne and Stein, 2007). The *F. graminearum* produces mycotoxins, accumulates in wheat grains, and is harmful to food quality, thus, posing a severe threat to human and animal health (Zhang et al., 2013; Xiu et al., 2021).

In China, the increasing grain output is the primary goal of developing a modern agricultural path with Chinese characteristics. Studies have shown that despite a large number of measures that have been taken to protect crops from the threat of diseases and pests, the increase in yield is accompanied by an increased loss in crop production due to biotic and abiotic stresses (Lobell and Field, 2007; Schäfer et al., 2009). The methods to increase crop yield should be reliable for producers and safe for consumers, as well as the environment. Traditionally, agrochemicals played a critical role in disease control, but this has led to an increase in environmental pollution and induced pesticide resistance (Zhang et al., 2017). Hence, using endophytes in the rhizosphere to develop biological defenses against plant diseases, as a safer and more productive means of agricultural practices, has been attracting more attention to researchers and scientists. Those plant growth-promoting rhizobacteria (PGPR) have been well-evaluated and concluded by Gouda et al. (2018). Compared with endophytic bacteria, the role, and usages of endophytic fungi for sustainable agriculture remain controversial and restricted.

Generally, as a beneficial companion in plants, endophytic fungi help plants to resist both biotic and abiotic stresses by regulating the immune response of plant-microbe interactions and stimulating the production of metabolites (Tedersoo et al., 2020; Rajani et al., 2021). Therefore, endophytes have a broad application prospect for use as biological control agents and biological fortifiers. However, there is still a long way to go to integrate endophytes into conscious agricultural production because not all endophytes are inherently useful, which is strongly dependent on plant species and genotypes (Lu et al., 2021). Therefore, the complex and dynamic communication relationship between parasitic crops and endophytic fungi needs to be clarified.

*Piriformospora indica* is a root-endophytic basidiomycete member of the order Sebacinales, which was first discovered by Indian scientists in the Thar desert in northwestern India in 1998 (Verma et al., 1998). The *P. indica* is well-known to be able to establish beneficial interactions with many different hosts, including monocotyledons such as barley, wheat, rice, corn, and dicotyledons, such as *Arabidopsis* and tobacco (Qiang et al., 2012). The *P. indica* serves as an excellent model for beneficial microbes since it: (1) is genetically transformable and culturable in axenic conditions (Zuccaro et al., 2009); (2) promotes plant growth and yield (Kundu et al., 2021); (3) enhances plant tolerance against abiotic stress (Li et al., 2021a); and (4) primes plants for disease resistance against biotrophic and necrotrophic fungi (Stein et al., 2008), oomycetes (Trzewik et al., 2020), and viruses (Fakhro et al., 2010).

Among these advantages, its disease resistance develops it into a good biological control fungus and has been widely used. Studies have confirmed that *P. indica* can enhance plants’ resistance against *Cochliobolus sativus* (Sahay and Varma, 2006) and can induce systemic resistance against Erysiphe in barley (Deshmukh and Kogel, 2007) and *Verticillium dahliae* in *Arabidopsis* (Sun et al., 2014). Rabiey and Shaw (2016) evaluated the inhibitory effect of *P. indica* against *F. graminearu*m, but did not elucidate the protective mechanism of *P. indica* to plants. Furthermore, Panda et al. (2019) showed that *P. indica* rapidly and effectively activates jasmonic acid/ethylene (JA/ET) mediated basic defense mechanisms against pathogen infection by inducing transcriptome reprogramming to change the expression of JA/ET-related genes. In addition, *P. indica* can induce changes in gibberellin (GA), salicylic acid (SA), and abscisic acid (ABA) hormone levels, thereby inducing host immunity of microbe-associated molecular model (MAMP) (Jacobs et al., 2011) and self-regulation of mutual-symbiosis (Ding et al., 2008; Waller et al., 2008). Roylawar et al. (2021) reported that *P. indica* primes onion response against stemphylium leaf blight disease. Colonization of *P. indica* enhances the complete signal transduction cascade, leading to systematic expression of host defense genes. Studies have also shown that the colonization of this fungus may increase the production of defensive secondary metabolites in plants, such as artemisinin (Arora et al., 2016), abricin (Prasad et al., 2013), trichroterpene (Das et al., 2014), and curcumin (Bajaj et al., 2014).

It follows, therefore, that *P. indica* itself is a member of a system that activates multiple defense signals in plants and, thus, provides durable defense against multiple pathogens. Harrach et al. (2013) showed that *P. indica* protects barley roots from the loss of antioxidant capacity caused by *Fusarium*. Pre-inoculation with *P. indic*a effectively prevented the decrease of ascorbic acid/oxidized ascorbic acid ratio and glutathione content caused by *F. culmorum* pathogens. Among them, the activities of key antioxidant enzymes increased about 35% in plant metabolism after *P. indica* colonization. The above results indicate that the colonization of *P. indica* can stimulate antioxidant enzymes in plants and destroy reactive oxygen species (ROS) in plant cells, thus, triggering the defense response and improving the resistance of plants to pathogens. In addition, *P. indica* can enhance the resistance of rice to sheath blight by reducing the content of H_2_O_2_ and increasing the activity of antioxidant enzymes (Nassimi and Taheri. 2017). Recent studies have shown that *P. indica* can increase the expression of resistance genes in potatoes, but does not increase the activities of ascorbate peroxidase, peroxidase, and superoxide dismutase (SOD) (Li et al., 2021b). The further study corroborates the idea that *P. indica* is not likely to affect the defense enzyme response by itself but has a role in modulating onion biochemical reaction to *Stemphylium vesicarium* infection (Roylawar et al., 2021). Those data implied that different signal transduction pathways are induced by *P. indica* in different hosts, and the in-depth mechanism remains to be elucidated.

In this study, the effects of *P. indica on* systemic resistance and basal immunity against sharp eyespot and root rot diseases in wheat were evaluated at the physiological, biochemical, and molecular levels. Also, the effects of *P. indica* on inducing wheat resistance to sharp eyespot and head blight were compared. With the *P. indica*, as an effective biocontrol agent, the potential for disease resistance was fully assessed.

## Materials and Methods

### Fungal Inoculums Preparation and Inoculation

*Rhizoctonia cerealis* and *F. graminearum* isolated from wheat plants showing the symptoms of sharp eyespot and root rot were used in this study. The isolates of *P. indica* was obtained from the Professor Karl-Heinz-Kogel of Justus-Liebig-University, Gieseen, Germany. The *P. indica* was growing on complex medium (CM) medium plates and incubated at 23°C until used. The chlamydospores of *P. indica* were collected by flooding the surface of the 21-day-old CM plate with sterile distilled water by a spatula. Chlamydospore suspension was filtered through sterile Miracloth and the concentration of chlamydospores was adjusted to 1 × 10^5^/mL using a hemocytometer (Nassimi and Taheri, 2017). The inoculum of *R. cerealis* was prepared using the method described by Qi et al. (2021). Briefly, at the tillering stage, the stem base of each plant was inoculated with toothpick fragments harboring well-developed mycelia of *R. cerealis*. The inoculated sites were covered with wet cotton to increase the humidity, which promotes *R. cerealis* infection. Conidia of *F. graminearum* were harvested as described previously (Gunnaiah et al., 2012). The *F. graminearum* isolate (obtained from Prof. Yang, Tianjin Academy of Agricultural Sciences, Tianjin, China) was maintained on potato dextrose agar (PDA) media. For spore production, cultures were grown on rye B agar media, under UV light and darkness, for 16 and 8 h, respectively, at 25°C. Macroconidia were harvested and the spore count was adjusted to 1 × 10^5^ macroconidia ml^–1^. Wheat spikelets were inoculated with 10 μl of spore suspension using a syringe with an auto dispenser. The inoculated plants were covered with moistened plastic bags to maintain a saturated atmosphere to facilitate infection, and the bags were removed 48 h post-inoculation (hpi).

### Plant Materials

Seeds of wheat (*Triticum aestivum* L.) were surface sterilized by 15% sodium hypochlorite for 8 m and rinsed thoroughly with sterile distilled water. The seeds were treated by two methods: (1) Seed was germinated on wet and sterilized filter paper in a Petri dish at room temperature, after germination, the roots of germinated seeds were immersed into the suspension of *P. indica* for 6 h at 23°C. (2) Seeds of wheat were directly immersed into the suspension of *P. indica* at 23°C and change the fresh suspension of *P. indica* every 8 h until the seeds were germinated. The above treatments’ seedlings were then transferred to cultivation trays containing sterilized sand (121°C and 15 psi for 1 h) under greenhouse conditions (28 ± 4°C; 14/10 h light/dark photoperiod). The seedlings were planted in the 15-cm diameter pots (five seedlings per pot) filled with a 1:1 (v:v) mixture of sand and soil, which were sterilized in three successive days at 121°C and 15 psi for 30 min. To guarantee the colonization of *P. indica* into the wheat root, the roots of wheat seedlings were irrigated with a 20 mL spore suspension of *P. indica* per day after being transferred into the soil. The plants used as controls were inoculated similarly with sterile distilled water containing 0.05% (v/v) between 20. Different time intervals (3, 5, 7, 10, 14, 21, and 35 days) between inoculation of *P. indica* and pathogen were set. The inoculated plants were kept at dark conditions at 85% relative humidity and 23°C for 48 h. Following disease assessment, root volume, wet and dry weight of the roots, and shoots were measured.

### Plant Disease Evaluation

For disease evaluation, each plant was carefully pulled out and washed. Sharp eyespot disease severity was graded into six classes based on the site and number of diseased spots (1, I ≤ 5; 2, 5 < I ≤ 15; 3, 15 < I ≤ 25; 4, 25 < I ≤ 35; and 5, I > 35) and the disease index (DI) was calculated, as described previously (Zhu et al., 2015). Fusarium head blight disease severity was graded into five classes based on the proportion of stem discoloration (0 = no discoloration; 1 = 1 to 25%; 2 = 26 to 50%; 3 = 51 to 75%; 4 = more than 75%; and 5 = dead plant) as described by Fernandez and Chen (2005).

### Antagonistic Activity Assay

The interactions between *P. indica* and *R. cerealis*, the *P. indica* and *F. graminearum* were identified methodically. The 5-mm diameter plug of *P. indica* was placed on a side of a CM plate and incubated at 23°C. After 14 days, the same size plug of *R. cerealis* or *F. graminearum* from the margins of 7-day-old culture was placed at the other side of the plate. The interaction of two fungi was investigated macroscopically and microscopically (Rabiey et al., 2015) for 5 and 8 days post-co-cultivation.

### Detection of *Piriformospora indica* in Colonized Wheat Roots

The *P. indica* colonization in wheat root was identified by staining the root fragments using chitin-specific dye WGA-AF 488 (Molecular Probes^1^) according to the method of Li et al. (2017). Root samples were then subjected to microscopic observation.

### Relative Water Content Measurement

The Relative Water Content (RWC) displays the amount of water fraction in plant leaves and can be estimated by using the following formula as described by Weatherley (1950): RWC = FW−DW/TW−DW × 100. The weight of first leaf segments of wheat from each treatment and control plant was measured at 21 days after inoculation with *R. cerealis* and *F. graminearum* as fresh weight (FW). Afterward, leaf samples were immersed in distilled water at room temperature for 24 h and were weighted as turgid weight (TW), and the weights of leaf segments dried in an oven (70°C for 48 h) were recorded as dry weight (DW).

### Electrolyte Leakage Determination

Cell membrane stability was estimated by the electrolyte leakage from crowns of wheat plants with different treatments. Wheat crowns without fungal inoculation were used as controls. Crown samples at 21 days after inoculation by the pathogen were washed three times by distilled water and then kept in 25 mL distilled water at room temperature for 24 h. The electrical conductivity of a solution (EC1) was calculated by a conductivity meter (Dingguo, Tianjin, China). Then, the solution containing the leaves was transferred to autoclave at 121°C for 20 m and after cooling to room temperature, the final electrical conductivity (EC2) was measured by a conductivity meter. Membrane stability index (MSI) percentage was measured by the following equation (Singh et al., 1992): MSI = 1−EC1/EC2 × 100.

### Histochemical Detection of H_2_O_2_

Production of H_2_O_2_ in wheat plants was investigated using 3, 3’- diaminobenzidine (DAB) staining. The leaf samples with different treatments and controls without any inoculation or treatment were obtained at different time points and floated in the DAB solution (1 mg mL^–1^ DAB-HCL, pH 3.8) overnight. Alcohol and glycerin at the ratio of 8:2 were used to decolorize the leaf segments. The DAB polymerization at the site of H_2_O_2_ accumulation was produced in a reddish-brown polymer, which was microscopically investigated (Olympus IX83, Shibuya, Japan).

### Protein Extraction and Antioxidant Analysis

Total protein extraction was done according to the method described by Kar and Mishra (1976). Leaf tissues (300 mg) were sampled at 0, 6, 12, 24, 48, and 72 hpi with *R. cerealis* for different treatments. The samples were ground in liquid nitrogen and homogenized in 3 mL of 100 mM potassium phosphate buffer (pH 6.8). The mixture was centrifuged at 14,000 *g* for 20 m at 4°C and supernatant was used as enzyme source. Soluble protein concentration was investigated using bovine serum albumin as a standard (Bradford, 1976). Guaiacol peroxidase (GPX) activity was determined using guaiacol as a hydrogen donor. The reaction mixture (1.18 mL) contained potassium phosphate buffer (100 mM, pH 6.8), guaiacol (10 mM), H_2_O_2_ (70 mM), and enzyme extract (10 μL), and absorbance of the mixture was recorded at 470 nm for 3 m (Chance and Maehly, 1955). The activities of catalase (CAT), superoxide dismutase (SOD), and peroxidase (POD) were calculated as described by Aebi (1984) and Chance and Maehly (1955). The GPX and CAT, SOD, and POD activities were expressed as μmol min^–1^ mg^–1^ protein.

### Total RNA Extraction

The *P. indica* irrigation lasted for 2 weeks before *R. cerealis* and *F. graminearum* infection. Samples were harvested after respective treatments as shown in Table 1 and subjectively subjected to RNA extraction. Ethanol precipitation protocol and CTAB-PBIOZOL reagent were used for the purification of total RNA from the plant tissue according to the manual instructions. Grind tissue samples about 80 mg with liquid nitrogen into powder and transfer the powder samples in 1.5 ml of preheated 65°C CTAB-pBIOZOL reagents. The samples were incubated by a Thermomixer for 15 m at 65°C to permit the complete dissociation of nucleoprotein complexes. After centrifuging at 12,000× *g* for 5 m at 4°C, the supernatant was added with 400 μl of chloroform per 1.5 ml of CTAB-pBIOZOL reagent and was centrifuged at 12,000× *g* for 10 m at 4°C. The supernatant was transferred to a new 2-ml tube that added 700 μl acidic phenol and 200 μl chloroform, followed by centrifuging 12,000× *g* for 10 m at 4°C. The aqueous phase was added with an equal volume of the aqueous phase of chloroform and centrifuged at 12,000× *g* for 10 m at 4°C. The supernatant was added an equal volume of supernatant of isopropyl alcohol and placed at −20°C for 2 h for precipitation. After that, the mix was centrifuged at 12,000× *g* for 20 m at 4°C and then remove the supernatant. After being washed with 1 ml of 75% ethanol, the RNA pellet was air-dried in the biosafety cabinet and was dissolved by adding 50 μL of diethylpyrocarbonate (DEPC)-treated water. Subsequently, total RNA was qualified and quantified using a NanoDrop and Agilent 2100 bioanalyzer (Thermo Fisher Scientific, MA, United States).

**TABLE 1:** Respective treatments for different sample.

Number	Treatments	Abbreviation	Repeats	*P. indica* pre-inoculation	Harvest at () dai
1	Mock	Mock	3	−	14^0^ + 14^0^
2	*Piriformospora indica*	Piri	3	+	14^pi^ + 14^0^
3	*Fusarium graminearum*	Fg	3	−	14^0^ + 14^fg^
4	*Rhizoctonia cerealis*	Rh	3	−	14^0^ + 14^rh^
5	*P. indica* + *F. graminirum*	Piri + Fg	3	+	14^pi^ + 14^fg^
6	*P. indica* + *R.cerealis*	Piri + Rh	3	+	14^pi^ + 14^rh^

*14^0^ stands for days irrigation with water; 14^fg^ stands for Fusarium graminearum infection for 14 days; 14^pi^ stands for days pre-inoculation of P. indica; 14^rh^ stands for Rhizoctonia cerealis infection for 14 days.*

### mRNA Library Construction

The mRNA Library were constructed using the samples in Table 1. The Oligo(dT)-attached magnetic beads were used to purify mRNA. Purified mRNA was fragmented into small pieces with fragment buffer at the appropriate temperature. Then, first-strand complement DNA (cDNA) was generated using random hexamer-primed reverse transcription, followed by second-strand cDNA synthesis. Afterward, A-Tailing Mix and RNA Index Adapters were added by incubating to end repair. The cDNA fragments obtained from the previous step were amplified by PCR, and products were purified by Ampure XP Beads, then dissolved in EB solution. The product was validated on the Agilent Technologies 2100 bioanalyzer for quality control. The double-stranded PCR products from the previous step were heated denatured and circularized by the splint oligo sequence to get the final library. The single-strand circle DNA (ssCir DNA) was formatted as the final library. The final library was amplified with phi29 to make DNA nanoball (DNB), which had more than 300 copies of one molecular, DNBs were loaded into the patterned nanoarray and pair-end 100 bases reads were generated on the BGIseq500 platform (BGI-Shenzhen, China).

### Quantitative Real-Time Polymerase Chain Reaction Analysis

Total RNAs (1 μg) from control and treatment were used to make cDNA using M-MLV Reverse Transcriptase (Takara, Beijing, Japan), according to the supplier’s protocol, respectively. After treatment with DNase I (Sigma, Osterode am Harz, Germany), the cDNA was used as a template for quantitative real-time polymerase chain reaction (qRT-PCR) to quantify selected mRNAs using specific primers. The expression level of the respective gene was determined by quantitative RT-PCR. Quantitative RT-PCR was measured by SYBR Green fluorescence method as described previously (Livak and Schmittgen, 2001). In brief, quantitative PCR (qPCR) experiments were conducted on a Light Cycler96 Fast real-time PCR system (Roche, Basel, Switzerland)). The reaction solution contains 2 × Ultra SYBR Mixture 10 μL, 100 ng cDNA template, 10 μM forward, and reverse primers. Wheat actin was used as the control, and all experiments were conducted with at least three technical replications. Amplification program was applied as the following steps: the first initial activation step was performed at 95°C, 5 min, then followed by 30 cycles (95°C for 20 s, 56°C for 35 s, 72°C for 35 s, and 65°C for 20 s). At the end of each cycle, melting curves were determined respectively to guarantee the amplification of a single PCR product. The primers used in this work were listed in Supplementary Table 1.

### Statistical Analysis

In this study, all data are expressed as the means ± SE and represent at least three independent biological experiments. The significance of differences was analyzed by using a one-way analysis of variance (ANOVA) with Duncan’s multiple range test.

## Results

### Interaction of *Piriformospora indica* With *Rhizoctonia cerealis* and *Fusarium graminearum* in Wheat

**FIGURE 1:**
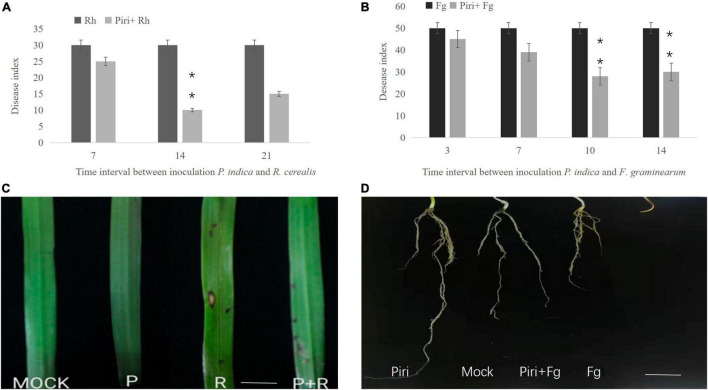
Determination of the best time interval between inoculating wheat seedlings with *Piriformospora indica* and *Rhizoctonia cerealis* or *Fusarium graminearum.* **(A)** Time interval between inoculation *P. indica* and *R. cerealis*; **(B)** Time interval between inoculation *P. indica* and *F. graminearum*. **(C)** Symptoms of sharp eyespot caused by *R. cerealis* on wheat leaves under different treatment (*P. indica* inoculation was 14 days before *R. cerealis* infection*)*; **(D)** Symptoms of root rot caused by *F. graminearum* on wheat roots under different treatment (*P. indica* inoculation was 10 days before *F. graminearum* infection*)*. Piri, *P. indica*; Fg, *F. graminearum*; Rh, *R. cerealis*; Piri + Fg, *P. indica* + *F. graminearum*; Piri + Rh, *P. indica* + *R. cerealis.*

We set different time intervals including 7, 14, and 21 days between the infection of *P. indica* and the *R. cerealis*, and different time intervals including 3, 7, 10, and 14 days between the infection of *P. indica* and the *F. graminearum* based on disease progression (Figure 1). The disease index was assessed after infection. We found that sharp eyespot developed most slowly on the wheat seedlings inoculated by the pathogen at 14 days post-inoculation (dpi) with *P. indica* (Figure 1A), whereas the lowest disease developing was observed on the wheat seedlings inoculation by *F. graminearum* at 10 dpi with *P. indica* (Figure 1B). The symptoms of damage caused by *R. cerealis* on leaves and caused by *F. graminearum* on roots were documented as shown in Figures 1C,D. The results indicated that pre-inoculation of spores of *P. indica* 14 days before *R. cerealis* infection could significantly reduce the eyesport number in the Piri + Rh treatment compared to the Rh (Figure 1C). And the pathogen of *F. graminearum* led to severe root rot, but this phenomenon could be remitted by *P. indica* pre-colonization 10 days before *F. graminearum* infection (Figure 1D).

### Effect of *Piriformospora indica* on Wheat Growth Under Pathogens Infection

**FIGURE 2:**
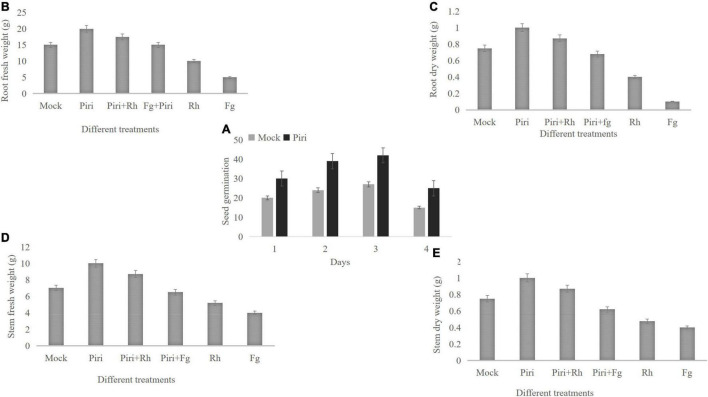
Effect of various treatments of Piri, Fg, Piri + Fg, Rh, and Piri + Rh on wheat seedlings growth parameters. **(A)** Seed germination number; **(B)** root fresh weight; **(C)** root dry weight; **(D)** stem fresh weight; **(E)** stem dry weight. Samples were treated as shown in Table 1 and harvested at 14 dpi with *F. graminearum* or *R. cerealis.* Piri, *P. indica*; Fg, *F. graminearum*; Rh, *R. cerealis*; Piri + Fg, *P. indica* + *F. graminearum*; Piri + Rh, *P. indica* + *R. cerealis.*

The seeds germination rate was calculated for those seeds directly immersed into the suspension of *P. indica.* Results indicated that pre-inoculation of *P. indica* before germination could significantly increase the rate of seeds germination (Figure 2A). Additionally, wheat seedlings inoculated by the pathogen at 14 dpi with *P. indica* were used to evaluate biomass production. The *P. indica* had a significant effect on growth characteristics of wheat seedlings such as root, stem fresh and dry weight of root, and shoot systems (Figures 2B–E). The biomass enhancement was obvious in wheat inoculated with *P. indica*, and pre-inoculation of *P. indica* significantly reduced the biomass loss caused by *R. cerealis* or *F. graminearum* (Figures 2B–E). In comparison, the side effects of *F. graminearum* on wheat yield were more serious than that of *R. cerealis*.

### Disease Index Evaluation and Relative Water Content and Membrane Stability Index Determination

**FIGURE 3:**
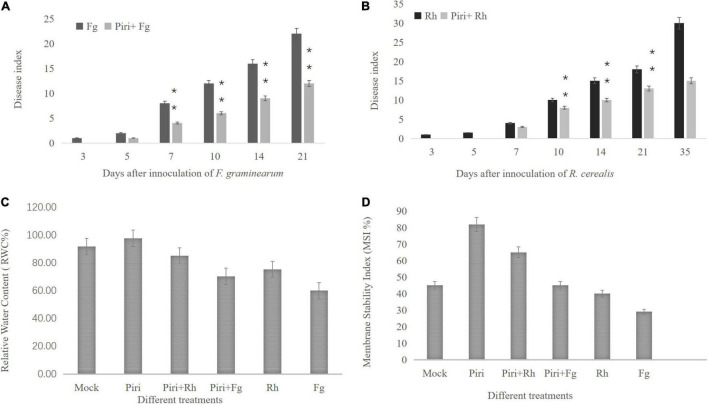
Disease index evaluation and relative water content (RWC) and membrane stability index (MSI) determination. **(A)** The wheat seedlings were infected by *F. graminearum* at 14 days post inoculation (dpi) with *P. indica* and harvested at different time-points, including 3, 5, 7, 10, 14, and 21 days. Effect of different treatments, including Fg, Piri + Fg on progress of the disease on the wheat seedlings were evaluated. **(B)** The wheat seedlings were infected by *R. cerealis* at 14 days post inoculation (dpi) with *P. indica* and harvested at different time-points, including 3, 5, 7, 10, 14, 21, and 35 days. Effect of different treatments, including Rh and Piri + Rh on progress of the disease on the wheat seedlings were evaluated. **(C)** Effect of various treatments of Mock, Piri, Fg, Piri + Fg, Rh, and Piri + Rh on relative water content (RWC). **(D)** Effect of various treatments of Mock, Piri, Fg, Piri + Fg, Rh, and Piri + Rh on membrane stability index (MSI). Piri, *P. indica*; Fg, *F. graminearum*; Rh, *R. cerealis*; Piri + Fg, *P. indica* + *F. graminearum*; Piri + Rh, *P. indica* + *R. cerealis.*

In this study, the effect of different treatments, including mock, *P. indic*a (Piri), *F. graminearum* (Fg), *R. cerealis* (Rh), *P. indica* plus *F. graminearum* (Piri + Fg), and *P. indica* plus *R. cerealis* (Piri + Rh) on the progress of the disease on the wheat seedlings were evaluated at different time-points (dpi) with *P. indica.* With the increase of infection time, the inhibition effect on the pathogen in wheat that was pre-colonized by *P. indica* gradually appeared. From the 7 dpi, the Piri + Fg treatment had a higher effect on reduction of disease severity caused by *F. graminearum*, and until 21 dpi, this inhibition effect was quite obvious. As for *R. cerealis*, from the 14 dpi, the Piri + Rh treatment had a higher effect on reduction of disease severity caused by *R. cerealis*, and until 35 dpi (Figures 3A,B).

In addition, RWC and cell MSI, by measuring electrolyte leakage of wheat crowns of plant leaves, were evaluated among various treatments, inducing mock, Piri, Piri + Rh, Rh, Piri + Fg, and Fg (Figures 3C,D). The obtained results revealed that RWC in plants inoculated with *P. indica* was highest among the six treatments. In addition, RWCs in Piri + Rh and Piri + Fg were higher than Rh and Fg’s, respectively, but lower than control. By contrast, the RWC in Rh was higher than in Fg (Figure 3C). On the other hand, the data trend of MSI was consistent with the RWC in the mock, Piri, Piri + Rh, Rh, Piri + Fg, and Fg (Figure 3D).

### Interaction of *Piriformospora indica* With *Rhizoctonia cerealis* and *Fusarium graminearum in vitro*

**FIGURE 4:**
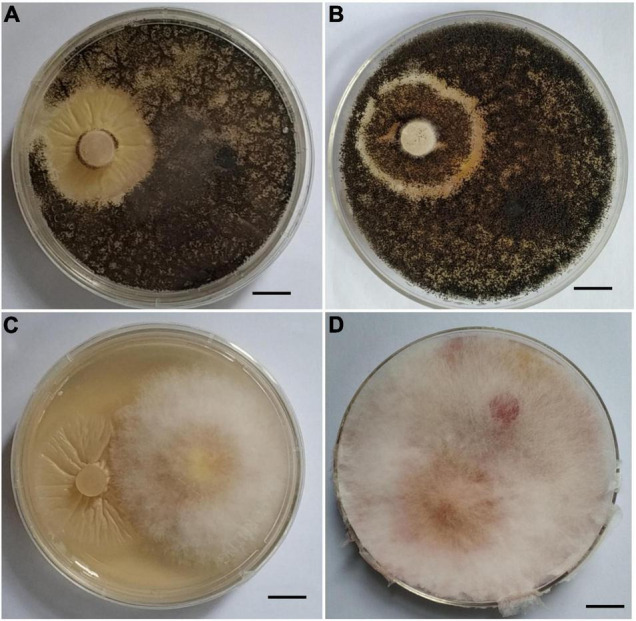
Interaction of *Piriformospora indica* with *Fusarium graminearum* or *Rhizoctonia cerealis* on PDA plates. **(A)** *P. indica* and *R. cerealis* grow on PDA plate at the 13 and 5th, respectively; **(B)** *P. indica* and *R. cerealis* grow on PDA plate at the 16 and 8th, respectively. **(C)** *P. indica* and *F. graminearum* grow on PDA plate at the 13 and 5th, respectively. **(D)** *P. indica* and *F. graminearum* grow on PDA plate at the 16 and 8th, respectively.

Since *P. indica* can inhibit pathogens’ infection in plants, we wanted to determine whether it can also inhibit pathogens’ infection *in vitro*. Co-cultivation of *P. indica* with *R. cerealis* or with *F. graminearum* on PDA plate indicated that the endophytic fungi had no antagonistic effect on them, and no obvious inhibitory zone was found at the meeting point of the two colonies (Figures 4A,B).

### Detection of H_2_O_2_ in Wheat Plants

**FIGURE 5:**
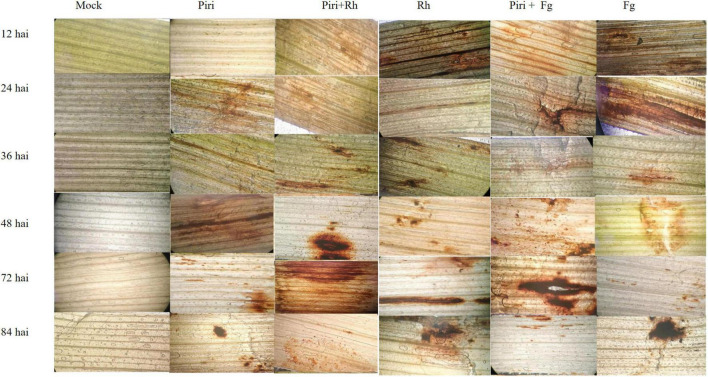
Detection of H_2_O_2_ in wheat leaf segments using 3, 3- diaminobenzidine (DAB) at various hours post inoculation under different treatments. Piri, *P. indica*; Fg, *F. graminearum*; Rh, *R. cerealis*; Piri + Fg, *P. indica* + *F. graminearum*; Piri + Rh, *P. indica* + *R. cerealis.*

The capabilities of six treatments, such as of mock, Piri, Piri + Rh, Rh, Piri + Fg, and Fg, for inducing H_2_O_2_ accumulation in wheat leaves, were investigated at 12, 24, 36, 48, 72, and 84 h, respectively, after inoculation (hai) (Figure 5). In the Piri + Rh group, the content of H_2_O_2_ reached the highest level at 48 hai, and then, gradually decreased. For the Rh, the content of H_2_O_2_ reached the peak level at 72 h after infection, which was later than that of the Piri + Rh group. Similarly, the content of H_2_O_2_ reached the peak after 72 h of infection in Piri + Fg treatment; whereas the highest level of H_2_O_2_ content was delayed to 84 hai in Fg treatment.

### Determination of Antioxidant Enzymes Activity

**FIGURE 6:**
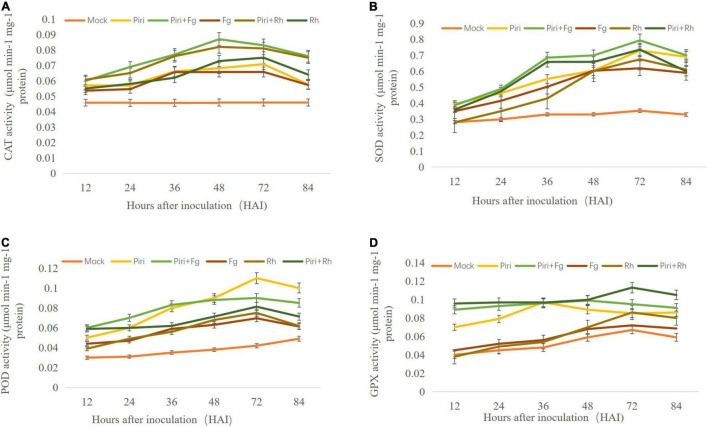
Determination of antioxidant enzymes activity in wheat seedlings with various treatments at different time points after different fungi inoculation. **(A)** Catalase (CAT) activity; **(B)** superoxide dismutase (SOD) activity; **(C)** peroxidase (POD) activity; **(D)** Guaiacol peroxidase (GPX) activity. Piri, *P. indica*; Fg, *F. graminearum*; Rh, *R. cerealis*; Piri + Fg, *P. indica* + *F. graminearum*; Piri + Rh, *P. indica* + *R. cerealis.*

To investigate the effect of various treatments on enzymatic antioxidants, activities of CAT, POD, SOD, and GPX were assayed in wheat seedlings at 12, 24, 36, 48, 72, and 84 hai, respectively (Figures 6A–D). The CAT activity in control plants without fungal inoculation had a stationary trend and was at the lowest level at all time points compared to other treatments tested. In Rh, CAT activity increased from 48 to 72 hai and then, decreased until 84 hai (Figure 6A). In Fg, the CAT activity increased from 36 hai. By contrast, the CAT activity in Piri + Rh, Piri + Fg was activated from 36 to72 hai and significantly higher than in Rh and Fg respectively. Also, CAT activity in the Pi treatment was higher than Rh and Fg, but lower than Piri + Rh and Piri + Fg.

The SOD activity in control plants exposed stationary estate and had the lowest level compared to other treatments tested. In Rh, SOD activity increased from 48 to 72 hai, followed by a decreasing trend until 84 hai (Figure 6B). In Pi + Rh treatment, an increasing trend of SOD activity was observed from 36 to 72 dai, and then, decreased. In Piri, a significant increase of SOD activity from 36 to 72 hai was observed, and then, displayed a stationary status until 84 hai. The highest level of SOD activity was observed at 48 hai in Fg treatment. In Pi + Fg treatment, an increasing trend of SOD activity was observed from 36 to 72 dai. The data indicated that pre-inoculation of *P. indica* spore could increase SOD activity either in Pi + Rh or Pi + Fg treatment from 36 to 72 dai, and then, remained approximately at the same level.

The POD activity in control plants without fungal inoculation had a stationary trend and was at the lowest level at all time points. In Rh and Fg, a similar trend was observed for the POD activity from 36 to 84 hai (Figure 6C). In Pi + Rh treatment, an increasing trend of POD activity was observed from 36 to 72 dai, and then, decreased. The POD activity in Pi + Fg has the same trend, but a higher POD activity was obtained than the Pi + Rh treatment. In Piri, significant increase of POD activity from 48 to 72 hai was observed. The highest level of POD activity was observed at 72 hai in Piri treatment. The data indicated that pre-inoculation of *P. indica* spore could increase POD activity either in Pi + Rh or Pi + Fg treatment from 36 to 72 dai.

The order of GPX activity in control plants without fungal inoculation had a stationary trend. In Rh and Fg, a similar trend was observed for the GPX activity from 36 to 84 hai (Figure 6D). By contrast, the GPX activity in Rh was a bit higher than in Fg treatment. However, In Piri, significant increase in GPX activity from 24 to 72 hai was observed. And the GPX activity in Pi + Rh treatment and Pi + Fg was higher than in Piri from 48 to 72 hai. In addition, Pi + Rh could promote higher GPX activity than Pi + Fg.

### Profile of Different Expressed Genes in Wheat Responsive to *Piriformospora indica* and Pathogens Colonization

The transcriptomes of wheat responsive to *P. indica* and pathogens colonization (see Table 1) were obtained and sequenced respectively by the BGI Seq500 platform. All the uni-genes were finally obtained by sequence splicing, a redundancy removal based on the sequence clustering software. Functional notation and cluster analysis of unigenes were performed by comparing unigenes to the database. Through data integration analysis, differentially expressed genes (DEGs) of wheat responsive to *P. indica* and pathogens colonization were analyzed (Table 2). The number of DEGs caused by root rot disease was 31,355, and the upregulation and downregulation genes were 17,859 and 13,496, respectively. The number of DEGs in wheat caused by sheath blight was 3,167, and the number of upregulated and downregulated genes was 1,988 and 1,179, respectively. However, *P. indica* had the least effect on the transcriptome of wheat, with only 641 DEGs, among which 147 genes were upregulated and 494 genes were downregulated, respectively.

**TABLE 2:** Differentially expressed genes (DEG) in the different comparison group.

	Comparison group	DEG number	Up expression of DEG	Down expression of DEG
Fg Group	Fg vs. Mock	31,355	17,859	13,496
	Piri vs. Mock	641	147	494
	Piri + Fg vs. Mock	27,516	17,142	10,374
	Piri + Fg vs. Fg	3,941	2,559	1,382
	Piri + Fg vs. Piri	20,757	11,467	9,290
Rh Group	Rh vs. Mock	3,167	1,988	1,179
	Piri + Rh vs. Mock	1,395	375	1,020
	Piri + Rh vs. Rh	2,126	541	1,585
	Piri + Rh vs. Piri	796	224	572

**FIGURE 7:**
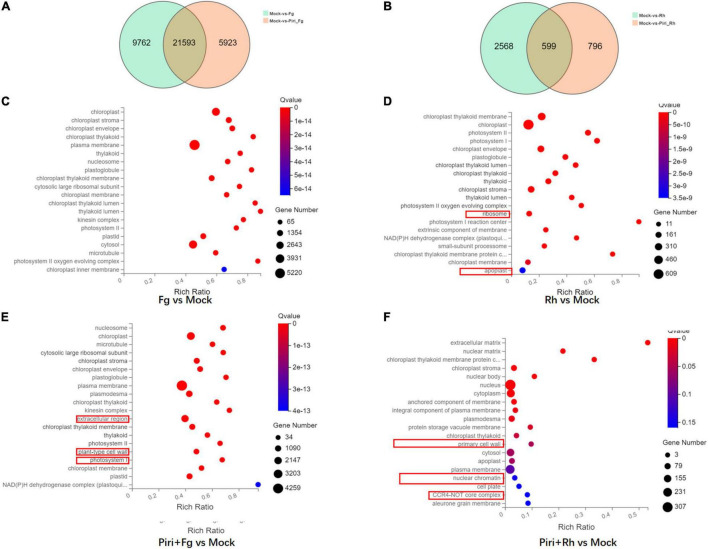
Profile of different expressed genes in wheat responsive to *P. indica* and pathogens colonization. **(A)** Venn Diagram of the different expressed genes (DEGs) between Fg vs. Mock and Piri + Fg vs. Mock. **(B)** Venn Diagram of the DEGs between Rh vs. Mock and Piri + Rh vs. Mock. **(C)** DEGs enriched in KEEG pathway in Fg vs. Mock group. **(D)** DEGs enriched in KEEG pathway in Rh vs. Mock group. **(E)** DEGs enriched in KEEG pathway in Piri + Fg vs. Mock group. **(F)** DEGs enriched in KEEG pathway in Piri + Rh vs. Mock group (/log2 FC/ > = 1, Q-value < = 0.05). Piri, *P. indica*; Fg, *F. graminearum*; Rh, *R. cerealis*; Piri + Fg, *P. indica* + *F. graminearum*; Piri + Rh, *P. indica* + *R. cerealis.*

It was shown that, compared to mock, Fg treatment produced 31,355 DEGs, by adding *P. indica*, the number of DEGs was reduced to 27,516 in Piri + Fg; whereas Rh treatment produced 3,167 DEGs and Rh + Piri produced 1,395 DEGs (Figures 7A,B). We can obtain some hints from the above data: (1) Transcriptome changes caused by *F. graminearum* were more severe than those caused by *R. cerealis*; (2) The number of DEGs in the transcriptome can be reduced by the addition of *P. indica*. The DEGs caused by *F. graminearum* reduced 18% and those caused by *R. cerealis* reduced 58% by the addition of *P. indica*. Additionally, the DEGs involved in the Kyoto Encyclopedia of Genes and Genomes (KEGG) pathway were analyzed (Figures 7C–F). In the Fg treatment group, the DEGs were mainly concentrated in the chloroplast-related pathways, including Chloroplast, chloroplast stroma, chloroplast envelope, chloroplast thylakoid, chloroplast membrane, and chloroplast inner membrane (Figure 7C). In the Piri + Fg group, besides the chloroplast-related pathways, DEGs in the plant Tpye cell wall, extracellular region, and photosystem I pathway were added, suggesting that these pathways probably play an important role in alleviating the effects of the disease (Figure 7E). Compared with the DEGs caused by *F. graminearum*, the DEGs caused by *R. cerealis* not only concentrated in the chloroplast-related pathway, but also involved in the ribosome and apoplast pathways, which implied that different pathways were activated by the two different pathogens (Figure 7D). By adding *P. indica*, another three pathways including the primary cell wall, Neclear chromatin, and CCR4-not Core complex pathway were added in the Piri + Rh group, and the defense role of these pathways also needs to be further explored (Figure 7F).

### Identification of the Critical Genes in the Key Pathway

**FIGURE 8:**
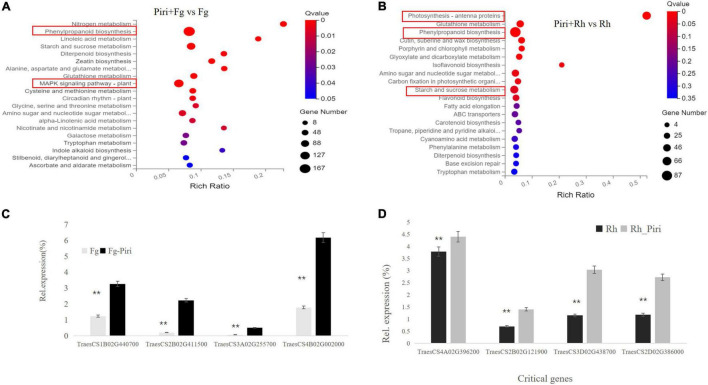
Differentially expressed genes (DEGs) analysis in Kyoto Encyclopedia of Genes and Genomes (KEGG) pathway and QPCR identification by comparing Piri + Fg vs. Fg and Piri + Rh vs. Rh. **(A)** DEGs analysis in KEGG pathway by comparing Piri + Fg vs. Fg; **(B)** DEGs analysis in KEGG pathway by comparing Piri + Rh vs. Rh; **(C)** Identification of the critical genes by QPCR in Piri + Fg vs. Fg group; **(D)** Identification of the critical genes by QPCR in Piri + Rh vs. Rh group. Piri, *P. indica*; Fg, *F. graminearum*; Rh, *R. cerealis*; Piri + Fg, *P. indica* + *F. graminearum*; Piri + Rh, *P. indica* + *R. cerealis.*

Because the addition of *P. indica* significantly alleviated the yield loss caused by *F. graminearum* and *R. cerealis*, we were curious to discover the molecular season of this difference at the transcriptome level. Significantly, DEGs were found in Piri + Fg vs. Fg comparison group (Figure 8A). There were 116 genes in the mitogen-activated protein kinase (MAPK) signaling pathway (plant), 71 of which were upregulated and 45 of which were downregulated. In the phenylalanine pathway, there were 167 DEGs, including 121 upregulated genes and 46 downregulated genes. In general, the number of upregulated genes was greater than that of downregulated genes in the Piri + Fg vs. Fg comparison group (Table 3).

**TABLE 3:** Differentially expressed genes (DEG) involved in the key KEGG pathway in the comparison group of Piri + Fg vs. Fg and Piri + Rh vs. Rh.

	KEGG pathway	DEG number	Up expression of DEG	Down expression of DEG
Piri + Fg vs. Fg	MAPK signaling pathway – plant	116	71	45
	Phenylpropanoid biosynthesis	167	121	46
Piri + Rh vs. Rh	Phenylpropanoid biosynthesis	87	17	70
	Photosynthesis – antenna proteins	53	0	53
	Starch and sucrose metabolism	48	11	37

In the Piri + Rh vs. Rh comparison group, significant DEGs were found mainly in phenylpropanoid biosynthesis, photosynthesis – antenna proteins, starch, and sucrose metabolism (Figure 8B). There were 87 DEGs, including 17 upregulated genes and 70 downregulated genes in the Phenylpropanoid biosynthesis pathway; In the fetish-antenna proteins pathway, 53 DEGs were downregulated. In Photosynthesis – antenna proteins pathway, there were 48 DEGs, 11 of which were upregulated and 37 of which were downregulated. In general, the number of downregulated genes was greater than that of upregulated genes in the Piri + Rh vs. Rh comparison group (Table 3). The significant DEGs in the KEGG pathway were explored and their expression levels were quantitatively analyzed by QPCR (Figures 8C,D). The functions of these genes and their accession numbers in National Care for Biotechnology Information (NCBI) were listed in Tables 4, 5. By comparing Piri + Fg vs. Fg, the significantly DEGs were functionally described as transcription factor WRKY19-like, ethylene-response factor C3/1B-like, mitogen-activated protein kinase kinase kinase 17-like, and were all upregulated in Piri + Fg (Table 4). In contrast, by comparing Piri + Rh vs. Rh, the significant DEGs were functionally described as WRKY transcription factor 70, LRR receptor-like serine/threonine-protein kinase, and cytochrome P450 84A1-like and were upregulated in Piri + Rh (Table 5).

**TABLE 4:** Identification of DEGs involved in the key pathway by comparing Piri + Fg vs. Fg.

Seq-ID	Gene functional description	NCBI accession No.
TraesCS1B02G440700	*Triticum aestivum* transcription factor WRKY19-like (LOC123147334)	XM_044566571.1
TraesCS2B02G411500	*Triticum aestivum* ethylene-response factor C3-like	XM_037633443.1
TraesCS3A02G255700	*Triticum aestivum* mitogen-activated protein kinase kinase kinase 17-like	XM_044485046.1
TraesCS4B02G002000	*Triticum aestivum* ethylene-responsive transcription factor 1B-like	XM_044516187.1

**TABLE 5:** Identification of DEGs involved in the key pathway by comparing Piri + Rh vs. Rh.

Seq-ID	Gene functional description	NCBI accession No.
TraesCS4A02G396200	*Triticum aestivum* WRKY transcription factor 72B-like	XM_044505189.1
TraesCS2B02G121900	Probable WRKY transcription factor 70	XM_044463411.1
TraesCS3D02G438700	Probable LRR receptor-like serine/threonine-protein kinase	XM_044495684.1
TraesCS2D02G386000	Cytochrome P450 84A1-like	XM_044477703.1

## Discussion

As an important worldwide food crop, wheat quality assurance plays an important role in maintaining the stability of food supply and food safety (Pehlivan-Karakas et al., 2021). However, wheat is often infected with many soil-borne diseases, resulting in reduced production. The sharp eyespot disease, caused by the necrotrophic fungus *R. cerealis*, and Fusarium head blight (FHB), caused mainly by the soil-borne fungus *F. graminearum*, are destructive diseases of wheat in many regions of the world (Gilbert and Tekauz, 2000; Hamada et al., 2011; Fernando et al., 2021). Therefore, the biological control capability of *P. indica* in wheat against sharp eyespot and FHB was investigated in this study.

In our effort, we have found that the germination rate of wheat seed that emerged in *P. indica* was significantly higher, and the germination time was 1 day earlier than the controls (soaked in water). In addition, our results proved that *P. indica* treatment was able to promote plant growth parameters, stimulating root biomass and fresh weight. Consistent with our data, colonization of *P. indica* has been confirmed to be able to increase the growth parameters of various plants (Deshmukh and Kogel, 2007; Waller et al., 2008; Sun et al., 2014; Nassimi and Taheri, 2017; Kundu et al., 2021; Liu et al., 2021), which undoubtedly makes us believe that *P. indica* can be considered as a biological fertilizer to improve plant yields. Several hypotheses have been formulated about the mechanisms of growth promotion of *P. indica*, which suggested that *P. indica* may actively promote auxin-regulated genes through the production of indole-3-acetic acid-like compounds (Lee et al., 2011; Franken, 2012). Of course, there are other possible reasons why *P. indica* promote plant growth. Studies have shown that root colonization by *P. indica* can hijack the ethylene signal pathway (Barazani et al., 2007). Because ethylene is used by plants to inhibit their growth under natural conditions, *P. indica* changes the ethylene pathway, which in turn may help promote host growth (Schäfer et al., 2009). A recent study indicated that Fe transporter (*PiFTR)* (Verma et al., 2021) and sulfate transporter (*SiSulT*) from *P. indica* (Narayan et al., 2021) play key roles in plant growth and development.

When we compare the difference between the treatment of *R. cerealis* and *F. graminearum*, it was clear that the disease effect of *F. graminearum* was more serious than *R. cerealis*. Previous physiological experiments have fully demonstrated that the colonization of wheat with *P. indica* can significantly inhibit the yield reduction caused by these two pathogens. In particular, we found that pre-soaking seeds in spore solution of *P. indica* could increase germination rates (Figure 1). These phenomena give us the confidence and motivation to unravel the mechanism behind them.

Our data demonstrated that dual culture on agar plate did not show any direct antagonistic effect of *P. indica* on *R. cerealis*, or *F. graminearum* (Figures 2A–D). These results were consistent with previous studies that *P. indica* has no resistance to the pathogen, including *F. graminearum*, *F. verticillioides*, *F. culmorum, Rhizoctonia solani*, and *Fusarium pseudograminearum in vitro* (Deshmukh and Kogel, 2007; Kumar et al., 2009; Rabiey et al., 2015; Nassimi and Taheri, 2017; Dehghanpour-Farashah et al., 2019). However, it has also been reported that *P. indica* can produce obvious inhibitory effects on *Verticillium dahliae* and *Gaeumannomyces graminis* on PDA plate (Ghahfarokhi and Goltapeh, 2010; Sun et al., 2014). This may indicate that the antagonistic effect of *P. indica* is species-specific *in vitro*.

*In vivo*, *P. indica* can inhibit disease effects caused by *R. cerealis and F. graminearum* (Figure 3). We found that a better antagonistic effect of *P. indica* would be produced *in vivo* when the time interval between *P. indica* and *R. cerealis* was 14 days (Figure 3B). As for *F. graminearum*, the best time interval between *P. indica* and *F. graminearum* was 10 days. And, in alleviating root rot, the role of *P. indica* is clear (Figure 3D). Previous evidence indicated that *P. indica* could induce resistance against *F. graminearum* and *F. culmorum* in barely (Deshmukh and Kogel, 2007; Waller et al., 2008; Harrach et al., 2013). Nassimi and Taheri (2017) reported that seedlings of rice pre-inoculated with *P. indica* showed a decreased level of disease caused by *R. solani*, which is consistent with our findings. Additionally, plants including barely, *Arabidopsis*, and maize pre-colonized *P. indica* significantly decreased the severity of the disease caused by *F. culmorum* and *Blumeria graminis* f. sp. Hordei, *Verticillium dahliae*, *F. verticillioides*, respectively (Waller et al., 2008; Kumar et al., 2009; Sun et al., 2014). Together with our data, this information strongly suggests that *P. indica* enhances plant resistance to pathogens through some indirect and complex mechanism.

Our study showed that RWC and MSI levels increased in the treatments of Pi and Pi + Rh compared to Fg/Rh and the control (Figure 4). The research demonstrated that electrolyte concentration in arbuscular mycorrhizal plants was higher than non-mycorrhizal plants because of the improved integrity and stability of membrane result from the colonization of arbuscular mycorrhizal fungi (Kaya et al., 2009). In addition, Naghashzadeh (2014) reported that the improved RWC and MSI were found in maize plants by applying mycorrhizal bio-fertilizers. The increased RWC and MSI in the *P. indica* colonized plant in our study implied that, like arbuscular mycorrhizal fungi, *P. indica* has the potential to be a biological fertilizer.

The results showed that both the disease of root rot and sheath blight could increase the content of H_2_O_2_ in leaves (Figure 5). This was consistent with previous studies, which have shown that soil-borne pathogens can accelerate plant cell death by increasing reactive oxygen species (ROS) and oxidative stress (Desmond et al., 2008; Cuzick et al., 2009). Our results further showed that the peak content of H_2_O_2_ appeared earlier in the Piri + Rh and Piri + Fg group, which indicated that the time point of hydrogen peroxide accumulation could be advanced by pre-inoculation of *P. indica.* In addition, our data showed that activity of SOD, POD, CAT, and GPX were activated in Piri, Piri + Rh, and Piri + Fg groups (Figure 6). Those data agreed with previously reported that *P. indica* can protect barley roots from loss of antioxidant enzymes caused by *F. culmorum* (Harrach et al., 2013). The research of Schäfer et al. (2009) and Jacobs et al. (2011) also clarified that *P. indica* intercepts MAMPs to induce immune responses, including oxidative bursts, induction of defense-related genes. Accordingly, it makes sense that the increased H_2_O_2_ content and enhanced antioxidant enzyme activity in plants pre-colonized by *P. indi*ca act as a signal to induce plants’ immune system, thereby enhancing plants’ resistance to pathogens. However, it is postulated that endophytes produce low levels of lytic enzymes as compared with pathogens, thus, avoiding triggering the plant immune response and gene products from mycorrhizal fungi are speculated to mimic the host cell signaling, thus, acting as decoys to circumvent plant defenses and gain entry into plant tissues (Trivedi et al., 2021). Obviously, *P. indica* acts as different patterns to participate in plant immune response. Overall, the interplay between plants and their endophytic microbiota is complex and still far from being fully elucidated.

Comparing the transcriptome of the different treatments, we found that transcriptome changes caused by *R. cerealis* were more intense than those caused by *F. graminearum* (Table 2). The growth promotion induced by *P. indica* was significant, although the transcriptome changes were not as large as those caused by the pathogen. Gene ontology (GO) analysis indicated enrichment in genes involved in various metabolic and catalytic processes. A previous study proved that the roots of the *Brachypodium distachyon* also displayed substantial transcriptional reprogramming following *P. indica* colonization (Šečić et al., 2021). These reprogramming genes must be responsible for the fungus-mediated growth promotion. In the Fg treatment group, the differential genes were mainly concentrated in the chloroplast-related pathways: Chloroplast, chloroplast stroma, chloroplast envelope, chloroplast thylakoid, chloroplast membrane, and chloroplast inner membrane. In the Piri + Fg group, different genes in the Tpye cell wall of the plant, in the extracellular region (1,733), and photosystem I pathway were added (Figure 7), suggesting that these pathways play an important role in alleviating the effects of the disease. The differential genes of sheath blight mainly concentrated in the pathways related to the chloroplast pathway, and involved in the ribosome and Apoplast pathways, suggesting that there were differences in the treatment pathways of the two strains. Compared with the Piri + Rh group, the differential genes of the primary cell wall, Neclear chromatin, and CCR4-not Core complex pathway were added, and the defense role of these pathways also needs to be further explored.

It was found that the addition of *P. indica* greatly alleviated the root rot caused by *F. graminearum* by comparing Piri + Fg to Fg treatment. Transcriptome sequencing showed that DEGs were mainly focused on the MAPK pathway and Phenylpropanoid biosynthesis (Figure 8A). Of them, 116 DEGs were found in the MAPK pathway, of which 71 were upregulated and 45 were downregulated. Accumulating data have demonstrated that MAPK cascades play central roles in plant immunity. In *Arabidopsis*, at least two complete MAPK cascades, the MEKK1-MKK1/MKK2-MPK4 and MAPKKK3/MAPKKK5-MKK4/MKK5-MPK3/MPK6 cascades, have been identified and reported to be involved in the elicitation of immune responses (Asai et al., 2002; Suarez-Rodriguez et al., 2007; Qiu et al., 2008; Yamada et al., 2016; Bi et al., 2018; Sun et al., 2018; Gao et al., 2021).

In rice, OsMAPKKK18 and OsMAPKKK24 also function downstream of receptor-like cytoplasmic kinases (RLCKs) and activate the OsMPKK4-OsMPK3/OsMPK6 cascade in the chitin signaling pathway (Kishi-Kaboshi et al., 2010; Wang et al., 2017; Yamada et al., 2017; Ma et al., 2021), suggesting similarity, as well as the diversity of MAPK cascades involved in immunity in different plant species. Therefore, we can determine that *P. indica* can participate in the activation of the plant immune system by activating the MAPK pathway. Thus, further experiments are warranted to determine which MAPK in wheat was involved in the elicitation of immune responses under the *P. indica* colonization.

On the other hand, we found that the phenylpropanoid biosynthesis pathway also participated in plant resistance to *F. graminearum*. Among 167 DEGs of phenylpropanoid biosynthesis pathway, 121 were upregulated and 67 downregulated. Plants have evolved exquisite mechanisms for the biosynthesis of secondary metabolites such as phenylpropanoids to cope with biotic and abiotic stresses (Dong and Lin, 2021). The significant DEGs in the phenylpropanoid biosynthesis pathway gave us reason to believe that *P. indica* can improve the wheat resistance to *F. graminearum via* regulating the phenylpropanoid biosynthesis.

In the case of *R. cerealis*, the pre-colonization of *P. indica* in wheat roots reduced the number of leaf spots caused by *R. cerealis* by comparing Piri + Rh to Rh treatment. Transcriptome sequencing showed that DEGs were mainly focused on phenylpropanoid biosynthesis, photosynthesis – antenna proteins, starch, and sucrose metabolism (Figure 8B). In the phenylpropanoid biosynthesis pathway, there were 87 differentially expressed genes, including 17 upregulated genes and 70 downregulated genes. In the photosynthesis – antenna proteins pathway, 53 differentially expressed genes were all downregulated. In the sucrose and sucrose pathway, the number of differentially expressed genes was 48, with 11 upregulated genes and 37 downregulated genes. By comparison, the responses of plants to the synergistic action of *P. indica* with the two respective pathogens were different. As reported, abiotic and biotic stresses widely reduce light-harvesting complex (LHC) gene expression in higher plants (Islam et al., 2020), which is consistent with our data that genes involved in Photosynthesis - antenna proteins pathway were all downregulated. However, control mechanisms and functions of these changes are not well-understood. Further research on the role of those defense components and their relationships with various types of reactive oxygen, antioxidant systems, in basal immunity will help us to design novel and effective disease management strategies to protect wheat plants against this destructive fungal pathogen.

## Conclusion

**FIGURE 9:**
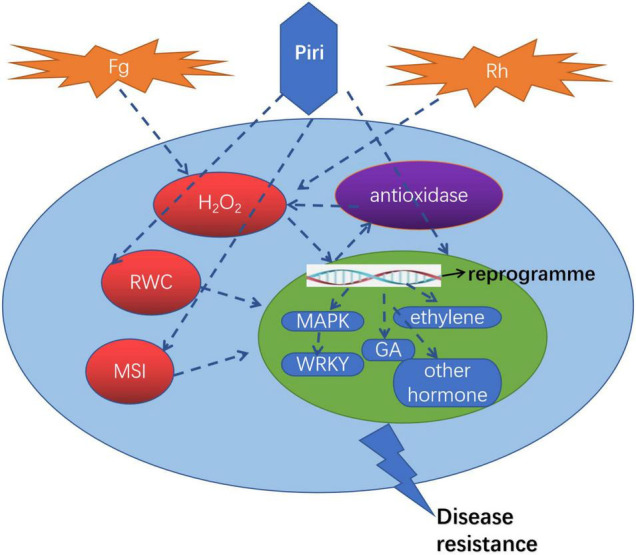
A proposed model illustrating the roles of *P. indica* in wheat against *R. cerealis* and *F. graminearum.* Piri, *P. indica*; Fg, *F. graminearum*; Rh, *R. cerealis*; RWC, relative water content; MSI, membrane stability index; GA, gibberellin acid; MAPK, mitogen-activated protein kinases.

A proposed model illustrating the roles of *P. indica* in wheat against *R. cerealis* and *F. graminearum* was drawn in Figure 9. The pathogens including *R. cerealis* and *F. graminearum* can induce the accumulation of H_2_O_2_ in plants, reduce the intracellular water content, and destroy the membrane stability. The addition of *P. indica* can reprogram the transcriptomic changes caused by *R. cerealis* and *F. graminearum*: (1) Inducing the expression of resistance-related genes, such as MAPK and WRKY; (2) Affecting the plant hormone signaling pathway; (3) Activating antioxidant pathway. Accordingly, wheat can directly resist the pathogen *via* the expression of resistance gene; Through the influence of the hormone metabolism pathway, *P. indica* colonization improved plant biological yield and enhanced plants tolerance to pathogens; By increasing the activity of antioxidant enzymes, the accumulation of reactive oxygen species in cells was decreased, and the water content in cells was further maintained and membrane stability was enhanced. Based on the above three modes, the resistance of wheat to these two pathogens was comprehensively improved.

## Data Availability Statement

The datasets presented in this study can be found in online repositories. The name of the repository and accession number can be found below: NCBI; PRJNA789469.

## Author Contributions

LL: conceptualization, methodology, investigation, formal analysis, visualization, and writing—original draft preparation, reviewing, and editing. NG and CL: investigation, formal analysis, and visualization. YF: software, data curation, and formal analysis. MD: software, investigation, and formal analysis. All authors approved the final manuscript.

## Conflict of Interest

The authors declare that the research was conducted in the absence of any commercial or financial relationships that could be construed as a potential conflict of interest.

## Publisher’s Note

All claims expressed in this article are solely those of the authors and do not necessarily represent those of their affiliated organizations, or those of the publisher, the editors and the reviewers. Any product that may be evaluated in this article, or claim that may be made by its manufacturer, is not guaranteed or endorsed by the publisher.

## Footnotes

1
www.invitrogen.com
