# Supplementary material for: Effect of Piriformospora indica-Induced Systemic Resistance and Basal Immunity Against Rhizoctonia cerealis and Fusarium graminearum in Wheat
Source: Front Plant Sci. 2022 Apr 14;13:836940. doi: 10.3389/fpls.2022.836940 (PMC9047502; doi:10.3389/fpls.2022.836940)
Supplement: Supplementary file 1 [file Table_1.docx]

Table S1. The primers used for QPCR identification

| Seq-ID | NCBI accession No. | Primer sequence |
| --- | --- | --- |
| TraesCS1B02G440700 | XM_044566571.1 | Forward5'-CAGAGAGCGCATATTCATGG-3'  Reverse5'-GCGGTGAACTGATGATGATG-3' |
| TraesCS2B02G411500 | XM_037633443.1 | Forward5'-GGCGACAAAACAAAAGGTCA-3'  Reverse5'-TTCAAAGCGAAACATTGACG-3' |
| TraesCS3A02G255700 | XM_044485046.1 | Forward5'-GTGGTGACCGACTCTTCCAT-3'  Reverse5'-ACTCCCATGTCGCTTCAAGT-3' |
| TraesCS4B02G002000 | XM_044516187.1 | Forward5'-GGTTTGGTTTGGTTTGGTGG-3'  Reverse5'-CGTCTGTATGTGCAGGAGGA-3' |
| TraesCS4A02G396200 | XM_044505189.1 | Forward5'-TCAGAAAGCAGGTGCAGAGA-3'  Reverse5'-AGGTCAGGTCAAGGGTGATG-3' |
| TraesCS2B02G121900 | XM_044463411.1 | Forward5'-GCCCTTTCATCAACATGGTC-3'  Reverse5'-TGATCGACTGGAAGCTGATG-3' |
| TraesCS3D02G438700 | XM_044495684.1 | Forward5'-TGACTTGAAGCCAAGCAATG-3'  Reverse5'-CCATCCACGACATGAACAAG-3' |
| TraesCS2D02G386000 | XM_044477703.1 | Forward5'-ACGAGGCAGAGACGGACTAC-3'  Reverse5'-CACTGATCTGGCAACACGAG-3' |
